# A study of genotypes, mutants and nucleotide sequence of hepatitis B virus in Pakistan: HBV genotypes in pakistan

**Published:** 2011-01-01

**Authors:** Khalid Mumtaz, Saeed Hamid, Shahid Ahmed, Tariq Moatter, Shamim Mushtaq, Anis Khan, Masashi Mizokami, Wasim Jafri

**Affiliations:** 1 Departments of Medicine and Pathology, Aga Khan University, Karachi, Pakistan; 2 Department of Clinical Molecular Informative Medicine, Nagoya City University, Nagoya, Japan

**Keywords:** HBV, Genotype, Hepatitis B, Epidemiology, Mutants, DNA, Pakistan

## Abstract

**Background:**

Hepatitis B virus (HBV) genotypes and mutations are gaining importance in determining the clinical course of chronic liver disease.

**Objectives:**

To determine and compare the distribution of HBV genotypes and genomic variations in Pakistan to other parts of the world.

**Patients and Methods:**

We conducted a prospective study at Aga Khan University Hospital from December 2006 to December 2008. HBV genotype was determined in 257 HBV DNA-positive patients. Patients were divided into two groups according to HBeAg positivity. Mutations in the pre-core and core promoter regions of HBV were determined in HBeAg-negative patients by line probe INNOLIPA assay.

**Results:**

The mean±SD age of patients was 28±5 years; there were 201 (78%) men. HBeAg was positive in 219 (85%) patients and negative in 38 (15%). HBeAg-positive patients were younger than HBeAg-negative patients (95% vs 21% in ≤30 years, p<0.001). HBV genotype D found in 247 (96.2 %) patients followed by a combined infection with HBV genotype B+D in 9 (3.3%) and 1 (0.5%) with genotype A. The mutations identified in 38 HBeAg-negative patients were T1762/A1764 in 21 (55.2%), PC mutant in 7 (18.4%), T1762/A1764/PC mutant in 2 (5%) and T1762/A1764/PC wild mutation in 1 (2%); no mutation identified in 7 (18.4%). Phylogenetic analysis did not show any significant differences between HBV genotype D isolated from Pakistan and those isolated from other parts of the world.

**Conclusions:**

HBV genotype D is predominant in Pakistan, irrespective of HBeAg status. PC and BCP mutations were found in significant numbers of patients infected with genotype D. The HBV genotype D isolates from Pakistan are identical to the sequences isolated from other parts of the world.

## Background

The complete nucleotide sequence analysis of hepatitis B virus (HBV) shows an inter-group divergence of >8%, and an intra-group divergence of <5.6% [1]. Based upon this inter-group divergence and complete nucleotide sequencing, HBV has traditionally been classified into eight genotypes (A to H) [2][3]. In addition to these acknowledged genotypes, there are putative genotypes that could not be classified into any of the above-mentioned groups; such variants include genotype I [4] and J [5][6]. HBV genotypes are known to have a distinct pattern of geographic distribution: Genotype A is found in North America and northern Europe, as well as in some parts of Africa [7], whereas genotypes B and C are common in Southeast Asia and genotype D is found universally [8]. Genotype E has been reported from western and South-east Africa [7]. Genotype F has been detected in South and Central America [7] and genotype G has been reported in France and North America [2]. The data on distribution of HBV genotype is still emerging from South-east Asia. The most predominant genotype reported from India is genotype D (around 79%), followed by C (30%) [9]; while in Iran genotypes D is reported as the most common HBV strains [10]. The expression of anti-HBeAb with the disappearance of HBeAg is accompanied by emergence of different types of mutants in the genome of HBV. The most common mutants are pre-core (PC) and basal core promoter (BCP) and their appearance is heralded by an increase in the viral load and virulence. Like HBV genotypes, HBV mutant species like the PC mutants also seem to have a particular distribution, being more frequent in geographic regions such as Asia and the Mediterranean basin, where genotypes B, C and D are predominant and are rare in North America and Europe, where genotype A is commonly found [11][12]. In studies from Europe, differences among HBV genotypes in the selection of BCP and/or PC mutations and disease progression have been demonstrated [13]. In the Asian context, studies provide strong evidence that HBV genotype B is associated with less active and insidious progressive liver disease compared with genotype C, but the basis for the difference in pathogenicity has not been determined. Further studies are needed to delineate the relation between other HBV genotypes and course and outcomes of HBV infection [14]. HBsAg, anti-HBe, and HBV DNA positive serological profile can also be related to mutations affecting the BCP, especially A1762T and G1764A. PC and BCP mutations are found in isolation or with each other; mostly these mutation are related to HBeAg-negative phenotypes. These mutations have been associated with active hepatitis, severe disease after liver transplantation, development of HCC and FHF [11][15]. There are studies from Pakistan reporting variable HBV genotypes and their frequencies; one study by Idress, et al, reported a predominance of genotype C, Alam, et al, found genotype D in 85%, Baig, et al, reported genotype D in 64% of patients [16][17][18] and Abbas, et al, reported genotype D as the predominant genotype; this study however, mostly included hemodialysis patients with HBV infection [19].

## Objectives

These studies are conducted on a local population with small sample size, and resulted in conflicting results and used less sensitive methods of genotyping. Henceforth, there is need to confirm these finding in a larger cohort. The objective of our study was to determine the country-wide distribution of HBV genotypes in Pakistan and the different HBV mutations in patients with HBeAg-negative infections. We also aimed at compare the nucleotide sequence of HBV isolates from Pakistan to those isolated from other parts of the world.

## Patients And Methods

### Patients

The study population consisted of 257 patients with chronic hepatitis B infection who were also HBV DNA positive, from all over the country diagnosed between December 2006 and December 2008. Blood samples were taken from patients who were selected by purposive convenient random sampling. The study samples were collected consecutively from various locations distributed widely in different parts of Pakistan so that they could reflect the distribution of HBV genotype in our country. The data on age, gender, HBeAg status and residential place of patients was included in the analysis to document the epidemiology of HBV mutants in the country.

### Serological and Biochemical analysis

The study population was tested for HBsAg, HBeAg, hepatitis B core IgM antibody (anti-HBc IgM) (Abbott EIA assay Chicago, IL, USA), HCV antibody and anti-HDV (Abbott Anti-Delta EIA assay, Chicago, IL, USA). Liver function tests including total bilirubin, alanine aminotransferase (ALT) and alkaline phosphatase levels, serum albumin and prothrombin time were checked using a fully automated analyzer. In our laboratory the upper normal limit for ALT was 33 IU/mL for women and 55 IU/mL for men.

### Nested PCR forDetection of HBV DNA

HBV DNA was extracted from 200 μL of serum using QIAmp DNA Blood Mini Kit (Qiagen, Inc., Hilden, Germany) and amplified for complete genome by previously reported primers [20]. Contamination-free environment was ensured by adequate physical separation of pre- and post-amplification areas. Prior to HBV RNA PCR assay, work bench was cleaned with DNA/RNA free wipes (Molecular Bioproducts, San Diego, CA, USA). Samples were then extracted and amplified in physically separate areas. Gloves were frequently changed during processing and aerosol barrier tips were used for setting up HBV RNA PCR assay. In addition, in each run no template controls were included for monitoring cross contamination.

### Genotyping of HBV

We used commercially available Line Probe Assays for HBV genotyping (INNOGENETICS, Belgium) [21]. To confirm the findings, PCR products of three randomly selected samples were sequenced directly for complete genome with the prism Big Dye (Applied Biosystems, Foster City, CA) in an ABI 3100 DNA automated sequencer. Sequences obtained were analyzed in both forward and reverse directions. Complete genomes were assembled using GENETYX version 11.0 (GENETYX, Tokyo, Japan). The sequences for phylogenetic analysis were retrieved from DDBJ/EMBL/GenBank corresponding to the accession numbers mentioned in the tree. Alignment was accomplished using CLUSTAL W and NJ tree was constructed with Tamura-Nei distance correction model.

### HBV Mutations

All patients with HBeAg-negative infection were analyzed for viral mutations in the PC and BCP regions. Commercially available Line Probe Assays for HBV mutation analysis (INNOGENETICS, Belgium) [22] was used for this purpose. INNO-LiPA Pre-Core kit detects mutations in PC codon 28, and BCP A1762T and G1764A. The kit can detect a wild type/mutant mixed population of circulating virus.

### Ethics

The study was approved by Ethical Review Committee, Aga Khan University Hospital, Karachi, Pakistan.

### Statistical Analysis

The data are presented as mean±SD. The data were analyzed by SPSS release 16 (SPSS, Chicago, IL, USA) using x2, Student's t tests and Fisher's exact test when appropriate. A p<0.05 was considered statistically significant.

## Results

### Demographic profile

The mean±SD age of patients was 28±7 years. Patients consisted of 201 (78%) men and 56 (22%) women (Table 1). HBeAg was positive in 219 (85%) patients suggesting they possibly had wild type of HBV infection; HBeAg was negative in 38 (15%). The large majority of patients with wild type of HBV infection (n=208; 95%) were <30 years of age compared to those with HBeAg-negative infection (n=8; 21%) (p<0.001). The mean serum ALT level was not statistically different in HBeAg-positive compared to HBeAg-negative patients (103 vs 96 IU/mL) (Table 1).

**Table 1:** Characteristic features of patients with HBV infections

**Characteristics**	**HBeAg Positive n=219**	**HBeAg Negative n=38**	**p-value**
**Male/Female**	170/49	30/08	NS a
**Mean age** (yr)	27	28	NS a
**<30 years**	155	08	<0.001
**>30 years**	64	30	
**Serum biliruin **(mg/dL)	1.2±0.9	1.3±0.7	NS a
**Serum ALT b**(IU/mL) (Mean±SD)	103±36	110 ±28	NS a
**Serum alkaline phosphates **(IU/mL) (Mean±SD)	101±12	92±07	NS a
**Serum albumin **(g/dL)	3.8±1.0	3.6±1.1	NS a
**Genotypes**			0.003
**D**	212	35	
**B+D**	07	01	
**A**	00	02	

^a^ NS: Not Significant

^b^ ALT: Alanine aminotransferase

### Hepatitis B virus genotypes

Of the 257 patients studied, there were 150 from Sind, 50 from Punjab, 37 from Baluchistan and 20 from North-west frontier province (NWFP). Of these, 247 (96%) had HBV genotype D infection and 9 (3.5%) had a mixed genotype B+D infection. There was only one patient with genotype A infection. Out of 247 patients with genotype D chronic hepatitis B infection, 217 (87.8%) had HBeAg-positive infection while 30 (12.2%) had a mutant infection. We were able to demonstrate that eight (89%) of nine patients with mixed B+D HBV infection had wild type of HBV infection and only one had mutant infection.

### Phylogenetic Analysis

**Figure 1:**
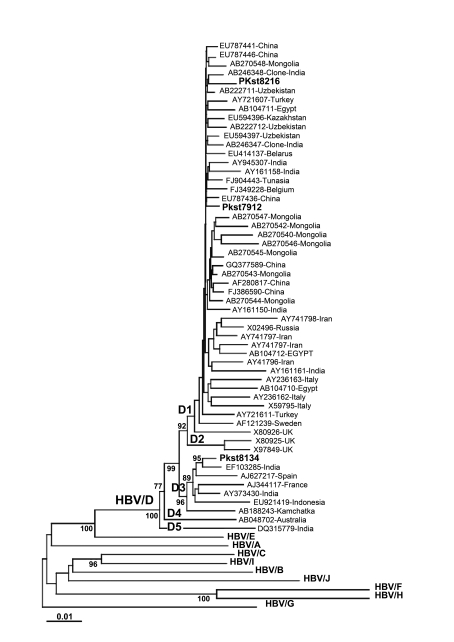
Phylogenetic tree of sub-genotypes of human HBV isolates from Pakistan.

Phylogenetic analysis revealed HBV genotype D (two with sub-genotype D1 and one with sub-genotype D3) in the three randomly selected samples, showing concordance with the genotyping results by Inno Lipa. Isolates did not segregate on phylogenetic analysis from the reference isolates in comparison, also suggesting that Pakistan isolates have the closest match from neighboring countries India and China in both D1 and D3 sub-genotypes (Figure 1). The nucleotide sequence data reported in this paper appear in the DDBJ/EMBL/GenBank nucleotide sequence databases with the accession numbers: AB583679, AB583680, and AB583681.

### HBV Mutations in HBeAg-negative patients

There were 38 patients with HBeAg negative status in our study group. Mutations were detected in 31 (81.5%) patients in different PC or BCP regions. The details of these mutations identified in the study are shown in Table 2. In analysis of three complete genomes for genomic variations, with the reference sequences retrieved from database, it was found that the case Pkst8216 (sub-genotype D1) had point mutations A1727G, T1653C and A1757G in addition to the BCP double mutation T1762/A1764 and PC stop codon mutation A1896. The same case also had a pre-S deletion of 48 nucleotides between 2999-3047. The other two cases did not show any specific genomic variations in comparison to the reference sequences in comparison.

**Table 2:** Details of pre-core (PC) and basal core promotor (BCP) mutations as detected by line probe assays in HBeAg-negative patients (n=38).

** Mutation **	** n (%)**
** PC mutations alone **	7 (18%)
** BCP T 1762 +A 1764 **	21 (55%)
** BCP T 1762 + A 1764 + PC mutant **	2 (5%)
** BCP T1762 +A 1764 + PC wild **	1 (3%)
** No mutation **	7 (18%)

## Discussion

We reported on a comprehensive country-wide report describing the prevalence of HBV genotypes and mutations in Pakistan. It is also confirming the phylogenetic analysis of hepatitis B reported by Baig, et al, [18] by full HBV genomic sequence from Pakistan. We found that genotype D was the predominant genotype in Pakistani patients with chronic HBV infection regardless of their HBeAg status. Mixed infection with genotypes B and D was present in a small percentage (3.5%) of patients; only one had genotype A infection. The mutation BCP A1762T and G1764A was found to be the most common mutation among HBeAg-negative patients. The knowledge and determination of HBV genotypes is gaining clinical and epidemiologic significance. There are reports suggesting that evolution of HBV into different genotypes probably started approximately 3000 years ago, by assuming the rate of nucleotide substitution at 1.4-3.2×105 per site per year. It is a known fact that the rate of nucleotide substitution per site per year remains almost constant, as long as the gene function remains unchanged [23]. Genotype D is considered to be the oldest type as it is originally widely distributed in the old world. High prevalence of genotype D may be associated with the old history of civilization in our region [24]. In addition to the age of a civilization, HBV belonging to the same genotype are believed to have an evolutionary relationship, and have been used for tracing the route of HBV transmission and geographical migration of subjects with chronic HBV infection. The uniformity of infection with genotype D virus suggests a possible similar source of infection in our patient population [24]. The importance of geographical distribution is further supported by the presence of similar genotype in the adjacent areas of Pakistan, i.e., northern India, where genotype D is reported in 46% of HBV patient population [25]. Furthermore, 92% of western India [26] and 75% of southern India has the similar genotype distribution [27]. We found mixed B+D HBV genotypes infection in nine patients, most of whom had wild type of HBV infection. Recombination between HBV genomes of two genotypes [22][28] and co-infection with more than one genotype may also be present in some patients. Genotypes of HBV have variable implications in selecting the patients for treatment. The results of studies with standard interferon (IFN) reported a favorable response to genotype C as compared to B (41% vs 15%) [29]. The results of HBV genotype for pegylated IFN are variable; Cooksley, et al, [30] showed improved response rate in genotypes B vs C (33% vs 21%); another study, however, did not find any difference (HBeAg seroconversion at the end of 24-week post-treatment follow-up) among viral genotypes: Genotype A (52%), genotype B (30%), genotype C (31%) and genotype D (22%) [31]. Nonetheless, overall, HBV genotype D is considered the least responsive of the major HBV genotypes to IFN therapy. The majority of studied patients in this study had HBeAg-positive infection; 15% had HBeAg-negative infection. This could be due to the fact that the majority of HBeAg-positive patients were young (<30 yrs of age); this figure could have been different in an older cohort. Almost all of our patients had more than one mutant infection except seven with PC codon 28 infection alone. Fifty-five percent of our patients had dual BCP A1762T and G1764A infection. It is reported that these mutants mostly produce chronic hepatitis followed by self-limited hepatitis or asymptomatic carrier status [32]. It is also reported that dual mutations can enhance the viral replication [28]. We used line probe assay for detecting mutations. This test was however, unable to detect any mutations in 18% of our patients. There is a possibility of detecting more mutants with the help of direct sequence analysis as shown by Davidson, et al. He compared the line probe assay with direct sequence analysis and found that it is more sensitive in detecting mixed infection in patients with genotype D [22][24]. Similarly, Maisa Ali, et al, compared INNO-LiPA HBV assay, direct DNA sequencing and subtractive PCR-RFLP or genotyping of clinical HBV isolates in 80 patients and found that the PCR-RFLP-based method incorrectly identified some genotype D strains [33]. Most of the previous studies reported from Pakistan have used PCR-RFLP methods and that is why they under-reported genotype D [16][17][18]. Our results indicated that genotype D is the predominant HBV genotype in Pakistan, and perhaps in South Asia, as indicated by studies from India. This should be kept in mind when considering treatment options for chronic HBV patients in this region. A considerable number of our patients with HBeAg-negative infection had PC and BCP mutations; mutations at multiple sites were common. Sequencing and phylogenetic analysis of HBV genotype D isolate in three of our patients was not different from HBV genotype D isolates from other parts of world.
